# Effect of floral cluster pruning on anthocyanin levels and anthocyanain-related gene expression in ‘Houman’ grape

**DOI:** 10.1038/hortres.2016.37

**Published:** 2016-08-10

**Authors:** Lei Zhang, Yan-shuai Xu, Yue Jia, Ji-yuan Wang, Yue Yuan, Yang Yu, Jian-min Tao

**Affiliations:** 1 College of Horticulture, Nanjing Agricultural University, Nanjing, P.R. China

## Abstract

Lateral floral clusters were removed from the main axis of the floral clusters of ‘Houman’ grape plants, leaving only 3–5-cm-long region of flowers at the end of the central axis. The floral clusters were pruned at 7 days prior to flowering. The effect of the pruning on fruit quality was assessed by determining the composition and levels of anthocyanins in the fruit and anthocyanin-related gene expression. Results indicated that floral cluster pruning significantly improved the quality of the fruit by increasing berry size, fruit weight and the total content of soluble solids. Floral cluster pruning also decreased the level of titratable acidity. Sixteen different anthocyanins were detected in fruit of the pruned clusters, while only 15 were detected in fruit from unpruned clusters. The level of anthocyanins was also significantly higher in fruit of the pruned clusters than in the unpruned clusters. Anthocyanin-related gene expression was also significantly upregulated to a higher level in fruit from pruned floral clusters as compared with unpruned clusters. The upregulation was closely associated with increases in anthocyanin biosynthesis.

## Introduction

Anthocyanins are important, red, blue, purple and purple-black plant pigments that are widely distributed in the cell vacuoles of flowers, fruits, roots, stems and leaves.^1,2^ Grape anthocyanins are mainly found in the skin of mature fruits. The degree and timing of fruit coloring depends on the type and level of anthocyanins that are synthesized. Skin color in grapes is an important index for evaluating fruit appearance and market value, and its importance to market prices has been gradually increasing. Anthocyanin synthesis occurs via the phenylpropanoid and flavonoid metabolic pathways. The genes involved in anthocyanin biosynthesis may be divided into two categories: structural genes, which are directly involved in anthocyanin biosynthesis,^3^ and regulatory genes such as *MYB* and *bHLH* that are not directly involved in anthocyanin synthesis but rather regulate the expression of anthocyanin structural genes.^4–6^ Genotypic differences among different varieties^7,8^ that influence anthocyanin synthesis and accumulation include sugar accumulation and hormone levels in the fruit,^9–11^ fruit maturation and development.^12^ Environmental factors and management practices that effect anthocyanin production include light, temperature, moisture, hormone treatments^13^ and root restriction.^14^ The interaction between the genotype, environment and management practices heavily influence the overall composition and levels of produced anthocyanins.

Floral pruning is a common practice used in the standard cultivation of grapes. It contributes to standardizing several parameters of each fruit cluster including the consistency of flowering, and final fruit quality. It also reduces the labor required to remove fruit. The traditional method of pruning grape entails the removal of the tip of the flower cluster. However, when using this approach considerable work is still required to remove the mature fruit, thus increasing production costs. A new pruning method, in which a 3~5-cm-long region at the end of the central axis of the floral cluster is left and the lateral axes are removed, have resulted in improved fruit quality in ‘Summer Black’ grape.^15^ In addition, it significantly increases the ratio of indole-3-acetic acid (IAA) to abscisic acid (ABA) and gibberellic acid (GA) to ABA, which was suggested to reduce flower and fruit drop and are beneficial to the growth and metabolic activity of floral clusters.^16^

Studies have shown that anthocyanin synthesis is influenced by hormones and root restriction,^14^ but the effect of floral cluster pruning on anthocyanin has not been fully examined. In the current study, ‘Houman’ grape plants, a hybrid breeding variety (*Vitis vinifera L.×V. labrusca L.*) that possesses a good appearance with violet-black fruit and strong disease resistance, was used to examine the effect of floral cluster pruning on fruit quality, the synthesis of anthocyanins and anthocyanin-related gene expression. The overall goal is to develop a theoretical basis for the beneficial effects of floral cluster pruning on the fruit quality of grapes.

## Materials and methods

### Plant material

This study was carried out in 2013 in a 3-year-old vineyard of ‘Houman’ (*V. vinifera L.×V. labrusca L.*) grape located at the Tangshan grape test site of Nanjing Agricultural University, Nanjing, China (32°03ʹ N, 118°46 E). The row spacing was 3.0×6.0 m. A completely random design was implemented which utilized 5 plants and 120 randomly selected floral clusters for 2 treatments (namely, pruned and unpruned (control) floral clusters). Each treatment included 3 biological replicates of 20 floral clusters. Only 3–5 cm at the tip of the central axis of each floral cluster was left after pruning. All of the subtending floral axes were removed 7 days prior to flowering.

### Berry growth and quality

Sixty berries were picked from different portions (lower, middle and tip) of each of 20 pruned and unpruned clusters and were analyzed for fruit size, total soluble solids (TSS) and titratable acidity (TA). The TSS/TA ratio was also calculated at different stages (60–88 days after anthesis (DAA)) of grape berry development and ripening. Ten berries were analyzed for each of the three biological replicates. Berry size was measured using an electronic vernier caliper. TSS were measured using an RA-250 refractometer (ATAGO Technologies, Tokyo, Japan). TA was measured using the NaOH method.

### Anthocyanin extraction and HPLC-ESI MS analysis

Twenty berries from ten clusters of each treatment (pruned and unpruned) were used as a biological replicate and each assessment utilized three biological replicates. Berries were collected at different times from veraison through harvest. The peels from the berries were stored at −70 °C.

Anthocyanin content was measured as described by Wang *et al.*^17^ with some modifications. The identity and quantification of individual anthocyanins was performed as described by He *et al.*^18^ with minor modifications. All instruments were controlled by a Chromeleon 1.0 Xcalibur 2.1 workstation (CA, USA).

The mobile phase of aqueous 0.1% formic acid (solvent A) and acetonitrile (solvent B) were employed for high-performance liquid chromatography (HPLC) analysis at a flow rate of 0.2 mL min^−1^. The following linear gradient was used (proportion of solvent B): 0–5 min, 10%; 5–40 min, 5–95%; 40–50 min, 95%; 50–52 min, 95–10%; and 52–60 min, 10%. A 5 μL shot volume was used each time and 525 nm was the wavelength used for detection. The positive ion was carried out on mass spectrometry (MS) pattern and the instrument parameters using an electrospray ionization (ESI) interface; 35 psi nebulizer pressure; using N2, 10 L min^−1^ drying gas and using 300 °C to dry the gas; and a scan range, 150–1200 *m/z*.

The described instrumentation and protocol enabled the identification of anthocyanins based on sample retention time, molecular and ion fragment weights, and published data.^19–23^ The instrumentation also permitted the recognition of different proportions of *cis*- and *trans*-isomers of different anthocyanins based on their retention time. The *cis*-isomers of coumaroylated anthocyanins were eluted in a reversed-stage HPLC column earlier than the *trans*-isomers, and were present in lower proportions in the analyzed grape berries; which is in agreement with previous studies.^19,22–24^ The anthocyanin content data is presented as the Cy-3-*O*-glucoside equivalent from mg per g berry fresh weight.

### RNA extraction and RT–qPCR

Total RNA was extracted from the skins of grape berries using the cetyltrimethylammonium bromide method with minor modifications..^25^ cDNA was synthesized using a Takara PrimeScript RT reagent kit (Takara Bio, Otsu, Japan) and reverse transcription–quantitative PCR (RT–qPCR) was performed with a 7300 Real-Time PCR System (Life Technologies, NY, USA). *KyActin1*^25^ was used as a house keeping gene for normalization. Primers for some of the genes were designed using Beacon Designer software (CA, USA). Primers pairs of *F3H2*, *F3'H*, *F3'5'H*, *DFR*, *LDOX* and *3GT* were based on the information provided in Jeong *et al.*^26^ Primers pairs used for *MYB5a* were based on the report by Deluc *et al.*^27^ The primers pairs used for *MYB5b* were based on the report by Deluc *et al.*^28^ All of the primer pairs utilized in the study are listed in Table 1. Gene expression in the pruned and unpruned floral clusters was calculated using the 2^−ΔΔCT^ method.^29^

## Results

### Berry development and quality

**Figure 1:**
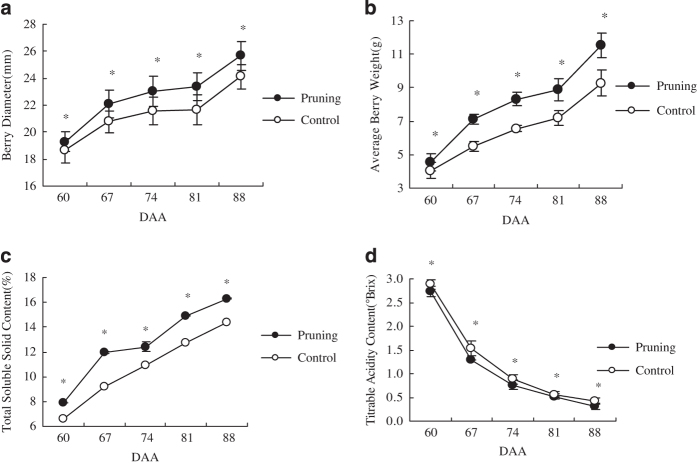
Effect of floral cluster pruning on berry diameter (**a**), weight (**b**), total soluble solid content (**c**) and titratable acidity content (**d**) at different stages of berry development (mean±s.e., *n*=30). '*' indicates a significant difference between treatments; *P⩽0.05.

Berry quality was measured from the onset of veraison (60 days after anthesis (60 DAA). Floral cluster pruning significantly increased the diameter of berries (Figure 1a). Berry weight was closely associated with berry size. The average weight of berries obtained from fruit clusters subjected to floral cluster pruning was 11.53 g, which was 1.2 times greater than berries from unpruned floral clusters (Figure 1b). The TSS value (Figure 1c) was also significantly higher in berries from pruned floral clusters throughout the entire ripening process, while TA (Figure 1d) was significantly lower in berries from pruned floral clusters during the entire sampling period, and was 70% of the controls (berries from unpruned clusters) at harvest.

### Anthocyanin content and composition

**Figure 2:**
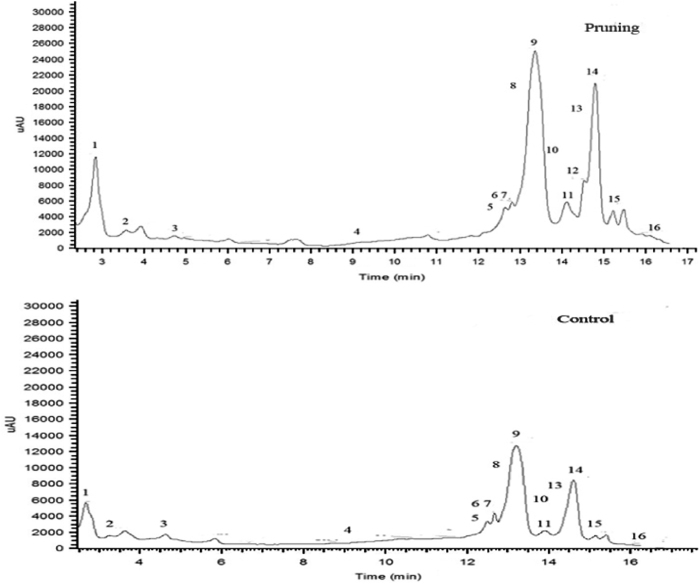
HPLC chromatograms of anthocyanidins in ‘Houman’ mature grape berries obtained from pruned floral clusters (pruning) and unpruned floral clusters (control). Different peaks represent different kind of anthocyanidins.

Using the spectra produced by HPLC-DAD and MS, 16 different anthocyanins were detected in the skin of ‘Houman’ grape berries (Figure 2). The fragment and molecular ions of these anthocyanins are listed in Table 2. There were three delphinidin (Dp) derivatives (peaks 4, 8 and 16); three cyanidin (Cy) derivatives (peaks 2, 6 and 11); three petunidin (Pt) derivatives (peaks 7, 12 and 13); four peonidin (Pn) derivatives (peaks 1, 3, 10 and 15) and three malvidn (Mv) derivatives (peaks 5,9 and 14). In contrast to the berries from the pruned floral clusters, Pt-3-*O*-(6″-*O*-coumaroyl)-glucoside was not detected in grape berries obtained from unpruned floral clusters.

**Figure 3:**
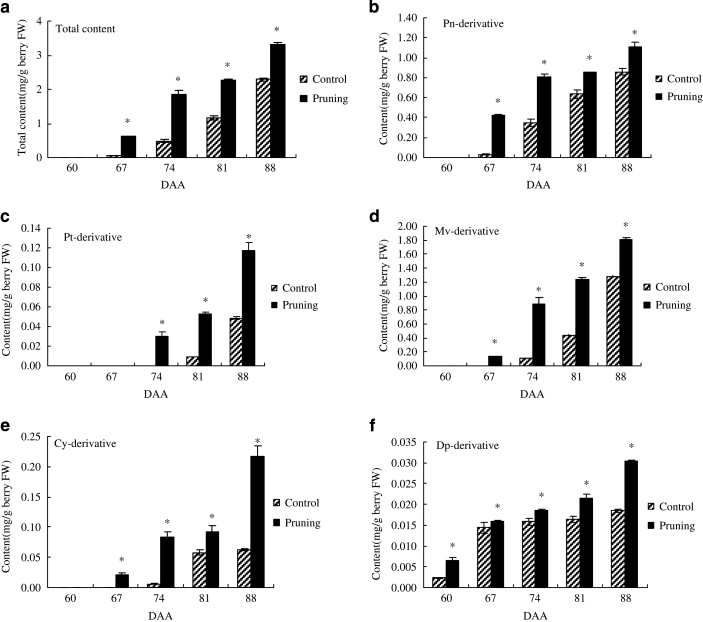
Changes in the concentration of total anthocyanins and five main antyocyanidin derivatives at different stages of ‘Houman’ grape berry development. Berries were obtained from pruned and unpruned (control) floral clusters. (**a**) Total content. (**b**) Pn-derivative. (**c**) Pt-derivative. (**d**) Mv-derivative. (**e**) Cy-derivative. (**f**) Dp-derivative. (mean±s.e., *n*=3). '*' indicates a significant difference between treatments; *P⩽0.05.

The concentration of total anthocyanin in both groups displayed a similar trend during berry development and maturation (Figure 3). Anthocyanins levels in harvested berries (88 DAA) were higher (3.31 mg g^−1^) in grape berries obtained from pruned floral clusters than in berries (2.2901 mg g^−1^) from unpruned clusters. Higher levels of total anthocyanins were detected in berries from pruned floral clusters than unpruned controls throughout the sampling period (*P*≤0.05). The anthocyanin concentration in the pericarp of 'Houman' grapes from pruned floral clusters also increased rapidly beginning at 67 DAA, while in the control did not begin to increase until 74 DAA. These data indicate that floral cluster pruning not only significantly promoted coloring but also advanced the onset of coloring. The timing of the synthesis of the five different groups of anthocyanin derivatives also varied. Only Dp derivatives were detected in grape skins at 60 DAA. Three of the other anthocyanin derivatives appeared gradually, and the Pt derivatives were the last to appear at 74 DAA. Mv derivatives were the most dominant type of anthocyanin present and the proportion of the total anthocyanidin derivative content was 54.66% in berries obtained from unpruned floral clusters and 55.91% in berries from the unpruned clusters at harvest (88 DAA). The remaining anthocyanin derivatives exhibited lower levels than the Mv derivatives, and in order of decreasing content were Pn>Cy>Pt>Dp.

### Analysis of anthocyanin-related gene expression

**Figure 4:**
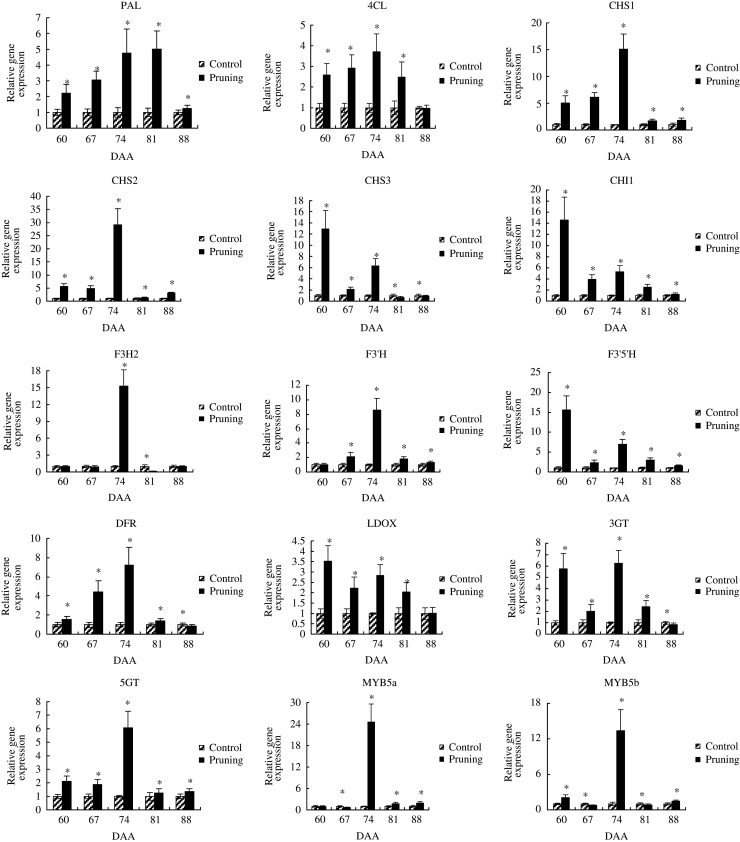
Relative expression of anthyocyanin biosynthesis-realted genes at veraison in grape berries obtained from pruned and unpruned (control) floral clusters. All control data set as 1. (mean±s.e., *n*=3). '*' indicates a significant difference between treatments; *P⩽0.05.

The profiles of anthocyanin-related gene expression in the pruned versus the unpruned floral clusters are shown in Figure 4. The relative expression of 15 genes of the anthocyanin biosynthesis pathway were assessed by RT–qPCR to determine if the expression values would explain the differences in anthocyanin levels in grape berries from pruned and unpruned floral clusters. All 15 genes were upregulated in pruned floral clusters from 60 DAA until ripening. Genes coding for PAL and 4CL, key enzymes of the phenylalanine metabolic pathway, exhibited similar trends in expression. The expression levels of *PAL* and *4CL* in grapes berries from pruned floral clusters were 5 times greater than in grape berries from unpruned clusters at 81 DAA and three times greater than the control at 74 DAA. The expression level of *CHS1*, *CHS2* and *CHI1* were also upregulated to a higher degree throughout the sampling period in berries from the pruned floral clusters than in berries from the unpruned floral clusters. *CHS3* was upregulated before 74 DAA but was downregulated at harvest. *F3’H* and *F3’5’H* code for enzymes that function at a branching point of the anthocyanin pathway, directing anthocyanin precursor to di- or tri-hydroxylated anthocyanins biosynthesis, which are then used to produce red Cy and blue Dp, respectively.^30^ *F3’5’H* and *F3’H* expression were both upregulated by the pruning treatment and remained upregulated throughout the sampling period. *F3H2* was the only gene that was downregulated, relative to the control, by the pruning of the floral clusters on nearly all of the sampling dates except 74 DAA. *DFR* was upregulated, relative to the control, in berries from the pruned floral clusters, except at harvest (88 DAA); while *LDOX* was upregulated on all of the sampling dates. Castellarin *et al.*^31^ reported that *3GT* gene expression was closely associated with anthocyanin concentration and that there was a cumulative effect on anthocyanin concentration from each different *GT* gene. The expression of *3GT,* like *5GT*, was also more highly upregulated in berries from pruned clusters, relative to the unpruned controls, on all sampling dates. The highest level of *3GT* and *5GT* expression was observed at 74 DAA, which may explain the continuously increasing level of anthocyanins that were measured. The transcription factors MYB5a and MYB5b exhibited a similar expression pattern, namely, lower at the beginning of veraison and at harvest, but higher at 74 DAA.

## Discussion

Methods to reduce fruit load in grapes include vine pruning, as well as blossom and fruit thinning. The traditional method of fruit thinning in grape includes the removal of some lateral branches and tip of the central axis.^32^ There are several disadvantages to this method, however, such as the over enlargement of some clusters, small berries, non-uniform ripening and low sugar content; all of which make it difficult to standardize quality and yields. Japan was the first production area to utilize the floral cluster pruning method, retaining only the 3–7 cm cluster tip in ‘summer black’cultivars. Jia *et al.*^15^ evaluated floral cluster pruning on ‘Houman’ grape, and results indicated that this approach helped to standardize production and was also convenient for bagging clusters, achieving uniform coverage of sprays and harvesting. Retaining only a 3-cm cluster tip, the current study examined the effect of floral cluster pruning on the composition and content of anthocyanins, relative to berries in unpruned floral clusters. The increase in the level of different anthocyanins also improved the appearance of fruit from pruned clusters as the color of the berries deepened.

**Figure 5:**
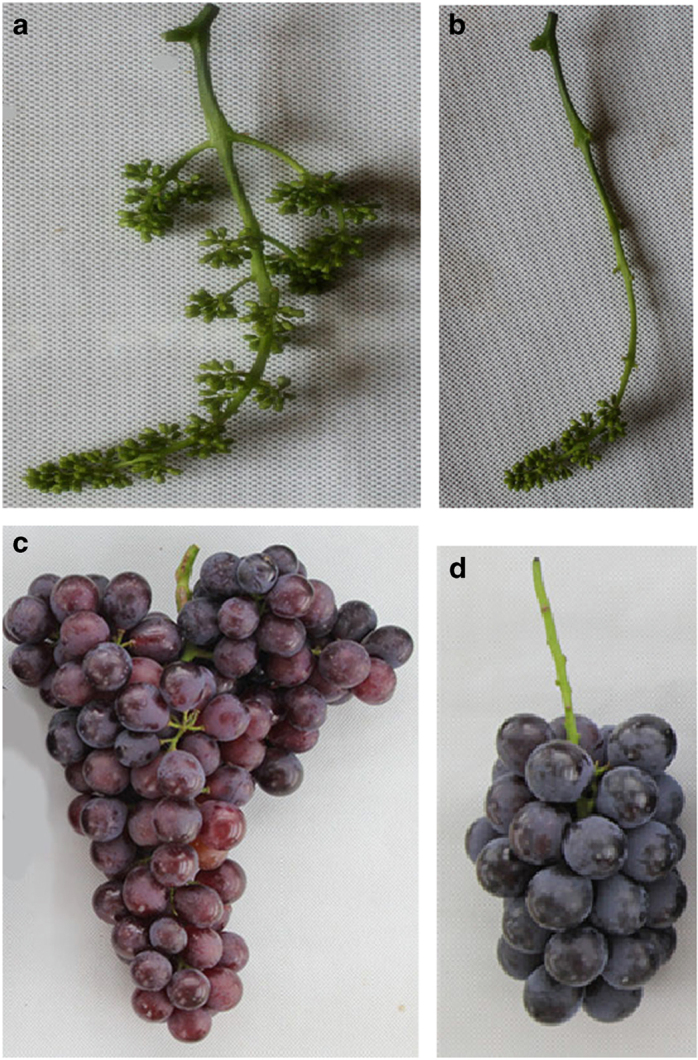
Unpruned (**a**) and pruned (**b**) floral clusters in ‘Houman’ grape and the resulting appearance of fruit from unpruned (**c**) and pruned (**d**) floral clusters.

The results of our study indicate that:

1. Floral cluster pruning significantly improved ‘Houman’ fruit quality by increasing the berry size, berry weight and TSS content, and by decreasing TA. In agreement with Jia *et al.,*^15^ the removal of all but 3, 5 or 7 cm of the cluster tip significantly increases berry size, berry weight and TSS content, and significantly decreases TA. Figure 5 illustrated the difference in the mature color of Houman’ grape berries obtained from unpruned and pruned floral clusters.
2. The color of the grape berries is a result of the composition and content of anthocyanins but the most decisive influence, however, is the genetic characteristics of the variety. There are numerous kinds of anthocyanins in grapes. Cantos *et al.*^33^ reported that the major anthocyanins in grapes were 3-*O*-monoglucosides of Pt, peonidin and malvidin, Dp, Cy, and that the main flavonols in the pericarp of ‘Crimson Seedless’ grape berries at ripening were quercetin 3-*O*-glucuronide and 3-*O*-glucoside. Five main anthocyanin glycosides (Dp, Cy, Pt, Pn and Mv) based on the position and number of benzene, hydroxyl and methoxy groups, are present in grape berries.^34^ Liang *et al.*^7^ detected 33 different kinds of anthocyanins in 110 varieties of grape, and reported that Mv derivatives were present at the greatest levels. On average, they represent up to 70% of the total anthocyanin Pn derivatives from the second greatest group. Similar results were observed in the present study. The concentration of total anthocyanins and individual anthocyanin derivatives increased significantly in ‘Houman’ grape berries as a direct result of floral cluster pruning. Mv derivatives compose up to nearly 55% of the total anthocyanins in berries from pruned clusters and 56% in the control berries from unpruned floral clusters. Wang *et al.*^14^ detected 29 different anthocyanins in Summer Black grape subjected to root restriction, which is 2 more than in their controls. In our study, 16 different anthocyanins were detected in berries of ‘Houman’ grape obtained from pruned floral clusters, as compared with 15 anthocyanins in berries of unpruned, control floral clusters. Our figure (Figure 2) indicated that Pt-3-*O*-(6″-*O*-coumaroyl)-glucoside was only found in the grape berries from pruned floral clusters. Collectively, Figure 2 indicates that floral cluster pruning mainly affected the overall level of anthocyanin in berry pericarp tissue but did not have a significant effect on the proportion of different anthocyanins present in the berries from pruned and unpruned floral clusters.
3. In general, the expression of structural, anthocyanin-related genes, including P*AL*, *4CL*, *CHI1*, *F3'H*, *F3'5'H*, *CHS1*, *CHS2*, *F3H*, *DFR*, *LDOX*, *OMT*, *3GT* and *5GT*, and anthocyanin-related regulatory genes, including *MYB5a* and *MYB5b*, were upregulated to a greater level throughout berry development and fruit ripening in berries derived from pruned floral clusters than in berries from pruned floral clusters. The upregulation of these genes was associated with a greater increase in anthocyanin biosynthesis in berries from the pruned floral clusters than in berries from the pruned floral clusters. As to the pattern of gene expression, one explanation is upregulation of the whole pathway by the action of transcription factors. Another potential reason for higher expression of all the genes tested is a structural change in the skin of the berries from the pruned clusters, for example, thicker skin and increased cell number. A previous study^3^ reported that Pt-3-glucoside biosynthetic pathway involves the following enzymes in the following order: *CHS*, *CHI*, *PAL*, *4CL*, *F3'5'H*, *F3H*, *DFR*, *LDOX*, *MT*. The study also reported that *F3'5'H* plays a key role in the synthesis of Pt-3-glucoside, while *F3'H* plays a key role in the biosynthesis of Cy-3-gulucoside and peonidin-3-glucoside. As illustrated in Figure 4, the expression of *F3'H* and *F3'5'H* at 60 DAA and 74 DAA are prominent, relative to other sampling times. *F3'5'H* was most highly expressed at 60 DAA, and so it is probable that Pt-3-glucoside was synthesized earlier than other anthocyanins. *F3'H* was most highly expressed at 74 DAA, so it can be inferred that 74 DAA is a key period for the biosynthesis of Cy-3-gulucoside and peonidin-3-glucoside. It should be noted, however, that *F3'5'H* was also expressed at that time.

## Conclusions

Floral cluster pruning significantly improved ‘Houman grape’ fruit quality and the accumulation of anthocyanins in the skin of the berries. Floral cluster pruning increased the concentration of individual anthocyanin derivatives and increased the number of anthocyanin derivatives present by 1 (a total of 15 in berries from unpruned clusters and 16 in pruned clusters). The majority of anthocyanin-related biosynthetic genes were upregulated to higher levels in berries from pruned floral clusters than from unpruned floral clusters. *PAL*, *4CL*, *CHS1*, *CHS2*, *CHI1*, *F3’H*, *F3’5’H*, *DFR*, *LDOX*, *3GT*, *5GT*, *MYB5a* and *MYB5b* were all upregulated to higher level in berries from pruned clusters, compared with the control, for all or part of the sampling period. The upregulation of these genes by floral cluster pruning was associated with an increase in anthocyanin biosynthesis.

## Figures and Tables

**Table 1:** Gene-specific primer sequences used in the RT–qPCR analyses

*Gene name*	*Accession no.*	*Forward primer*	*Reverse primer*
KyActin1	AB073011	5′-GATTCTGGTGATGGTGTGAGT-3′	5′-GACAATTTCCCGTTCAGCAGT-3′
PAL	JN858957	5′-CATCGAACGGGAGATCAACT-3′	5′-TGATGGCAGTCCATTGTTGT-3′
4CL	JN858959	5′-ACCACCTCCCTCTCCACAC-3′	5′-GCTCCGAGAAAGGAGAACG-3′
CHS1	AB015872	5′-CAGGCAGACTACCCGGATT-3′	5′-ACAGACGTTGGGGTTCTCC-3′
CHS2	AB066275	5′-ACCCACCTGGATTCTCTCG-3′	5′-GAAGGCTTCCACCAAGCTC-3′
CHS3	AB066274	5′-TGTGGATCAAAGCACCTATCC-3′	5′-TGGGGTTCTCTTTCAGGATCT-3′
CHI1	X75963	5′-AGACTGTGGAGGAGTTAGCG-3′	5′-AGAATGGAGTTGCCTGGTG-3′
F3H2	VitiA130	5′-CTGTGGTGAACTCCGACTGC-3′	5′-CAAATGTTATGGGCTCCTCC-3′
F3′H	AB213602-5	5′-GCCTCCGTTGCTGCTCAGTT-3′	5′-GAGAAGAGGTGGACGGAGCAAATC-3′
F3′5′H	AB213606	5′-AAACCGCTCAGACCAAAACC-3′	5′-ACTAAGCCACAGGAAACTAA-3′
DFR	X75964	5′-GAAACCTGTAGATGGCAGGA-3′	5′-GGCCAAATCAAACTACCAGA-3′
LDOX	X75966	5′-AGGGAAGGGAAAACAAGTAG-3′	5′-ACTCTTTGGGGATTGACTGG-3′
3GT	AF00372	5′-GGGATGGTAATGGCTGTGG-3′	5′-ACATGGGTGGAGAGTGAGTT-3′
5GT	GU237133	5′-TTCCATGGCTGAACTCAAAAC-3′	5′-AACATCCAACTGCTTGGTGAC-3′
MYB5a	AY555190	5′-CATGTCTCCCTGAAAATGATGA-3′	5′-TGCAAGGATCCATTTCACATAC-3′
MYB5b	AY899404	5′-GGTGTTCTTTAATTTGGCTTCA-3′	5′-CACAACAACACAACCACATACA-3′

Abbreviation: RT–qPCR, reverse transcription–quantitative PCR.

**Table 2:** Anthocyanins detected in ‘Houman’ grape berries

*Peak*	*Anthocyanidins*	*Retention time*	*[M]+ (m/z)*	*Frag.MS2 (m/z)*
1	Peonidin-3,5-*O*-diglucoside	2.82	625	301 463
2	Cyanidin-3-*O*-glucoside	3.21	449	287
3	Peonidin-3-*O*-glucoside	4.73	463	301
4	Delphinidin-3,5-*O*-diglucoside	9.12	627	465 303
5	Malvidin-3-*O*-(6ʺ-*O*-acetyl)-glucoside	12.31	535	331
6	Cyanidin-3-*O*-(6ʺ-*O*-coumaroyl)-glucoside,5-*O*-glucoside	12.75	757	595 449 287
7	Petunidin-3-*O*-(6ʺ-*O*-coumaroyl)-glucoside,5-*O*-glucoside	12.75	787	625 479 317
8	Delphinidin-3-*O*-glucoside	12.91	465	303
9	Malvidin-3-*O*-(6ʺ-*O*-coumaroyl)-glucoside,5-*O*-glucoside	13.36	801	639 493 331
10	Peonidin-3-*O*-(6ʺ-*O*-coumaroyl)-glucoside,5-*O*-glucoside	13.52	771	609 463 301
11	Cyanidin-3-*O*-(6ʺ-*O*-coumaroyl)-glucoside	14.00	595	287
12	Petunidin-3-*O*-(6ʺ-*O*-coumaroyl)-glucoside	14.35	625	317
13	Petunidin-3-*O*-glucoside	14.45	479	317
14	Malvidin-3-*O*-(6ʺ-*O*-coumaroyl)-glucoside	14.80	639	331
15	Peonidin-3-*O*-(6ʺ-*O*-coumaroyl)-glucoside	15.12	609	301
16	Delphinidin-3-*O*-(6ʺ-*O*-coumaroyl)-glucoside	16.45	611	303
